# Intimate partner violence among Arab women before and during the COVID-19 lockdown

**DOI:** 10.1186/s42506-021-00077-y

**Published:** 2021-06-16

**Authors:** Nessrin A. El-Nimr, Heba M. Mamdouh, Amal Ramadan, Haider M. El Saeh, Zeinab N. Shata

**Affiliations:** 1 Department of Epidemiology, High Institute of Public Health, Alexandria University, 165 El-Horrya Ave. El-Hadara, Alexandria, Egypt; 2 Department of Family Health, High Institute of Public Health, Alexandria University, Alexandria, Egypt; 3 Department of Health Education and Promotion, Jazan University, Medical Camp, Jazan, Kingdom of Saudi Arabia; 4 Faculty of Medicine, University of Tripoli, Tripoli, Libya

**Keywords:** Intimate partner violence, COVID-19, Pandemic, Lockdown, Arab, Women

## Abstract

**Introduction:**

Intimate partner violence (IPV) remains a serious human rights violation and an important health concern during the ongoing COVID-19 pandemic. The study aims to estimate the proportion of IPV among adult Arab women before and during the COVID-19 lockdown and to identify its possible predictors during the lockdown.

**Methods:**

A cross-sectional study was conducted between April and June 2020 using an online questionnaire. The sample included 490 adult Arab women aged 18 years and above, who live with their husbands. Data was collected using a Google forms designed questionnaire that included the socio-demographic characteristics, nature of lockdown, and exposure to different types of IPV before and during COVID-19 lockdown and the frequency of their occurrence. McNemar’s test was used to determine differences in the exposure to IPV before and during the lockdown, while logistic regression analysis was performed to identify the predictors of exposure to IPV during the lockdown.

**Results:**

Half of women reported that they were ever exposed to IPV with psychological violence ranking 1st. Exposure to any type of IPV and exposure to psychological, physical, and sexual violence have significantly increased during the lockdown compared to before the lockdown. The frequency of exposure to the different types of IPV ranged from 1–3 times per month to almost every day, but the most commonly reported was 1–3 times per month. Predictors of exposure to IPV during the COVID-19 lockdown included country of residence, family income, and whether the husband lost his job during lockdown.

**Conclusions:**

IPV has increased during the COVID-19 pandemic lockdown in the Arab countries, and it was associated with the socioeconomic consequences of the pandemic on families. Actions towards raising awareness about the problem among professionals and the community, early detection, and provision of appropriate services are mandatory.

## Introduction

Intimate partner violence (IPV) is a serious public health problem at the global level, with differences in its nature and occurrence [1]. Substantial amount of evidence related to IPV comes from Western countries, while there is still a scarcity of evidence of IPV in the Arab countries [2, 3]. Worldwide, 30% of women who have been in a relationship reported that they have experienced some form of IPV in their lifetime. The proportion of ever-partnered women, who reported any form of IPV in the Eastern Mediterranean region was higher than the world average (37%) [3]. The health impacts of IPV on women are significant as it can result in injuries and serious physical, sexual and reproductive health problem [2].

The public health emergency of the COVID-19 pandemic has brought to the surface the close link between crises and the potential increase in risk for IPV [4, 5]. The measures put in place to address the pandemic such as social distancing, staying home, and using containment measures could increase the likelihood of IPV. Pandemic-related risk factors include health and financial stresses at home, unemployment, reduced income, decreased access to services and ability to leave an abusive home, and the interruption of social networks including families and friends. Furthermore, the picture is compounded by confinement of women within the homes with abusive partners who used to practice coercive control mechanisms to exercise power over them [6].

Evidence from the USA, Australia, Brazil, Italy, and China indicated an increase in IPV reporting linked to the quarantine [7–10] with several western countries reported witnessing a surge in domestic abuse incidents that exceeded 21% to 35% than that of the previous year [11, 12].

Intimate partner violence remains a serious human rights violation and an important health concern during the ongoing pandemic. With the current lack of data on the impact of the COVID-19 pandemic on the IPV in Arab countries, addressing it became a priority. This study aims to estimate the proportion of IPV among adult Arab women before and during the COVID-19 lockdown and to identify its possible predictors during the lockdown.

## Methods

A cross-sectional web-based survey was conducted between April and June 2020 among adult (18 years and above) currently married women living with their husbands, Arab residents, and having basic Arabic reading and writing fluency skills. The sample size was calculated using Epi-info 7 software. Based on a prevalence of IPV of 37% in the World Health Organization (WHO) Eastern Mediterranean region [13], and 5% confidence limits, the minimum required sample size at 97% confidence level was 439 women. Study participants were recruited to the web-based survey via different advertisements, social media platforms including Facebook and WhatsApp, web forums frequented by married women, and email invitations. The responses were consecutively recorded until the completion of the required sample size. Out of 522 responses received, 490 forms with complete data were included in the final analysis.

Data were collected using a web-based 30-question survey. The questionnaire was designed using Google forms. The first section of the questionnaire included the socio-demographic data including age, level of education, and occupation of participants, the husband’s occupation, country of residence, and family income. The second section included questions about the nature of lockdown (partial or complete), whether the family income was affected by the lockdown, whether the husband lost his job during the lockdown, and the length of stay of husbands at home. The third section included questions about whether women were ever exposed at any time during her marriage to IPV (yes/no question) and the type of IPV they were exposed to (multiple response question). Exposure to different types of IPV before and during COVID-19 lockdown and the frequency of their occurrence during the lockdown were assessed by a series of 21 questions. These included exposures to verbal and psychological violence, physical and sexual violence, and financial abuse. The questionnaire also collected data regarding perceived reasons for the IPV and its consequences.

Each type of violence was assessed using three question: “Where you exposed before the lockdown?” (yes/no question), “Where you exposed during the lockdown?” (yes/no question), and “What is the frequency of exposure during the lockdown?”.

Verbal and psychological violence were defined as the use of verbal and non-verbal communication by the husband with the intent to harm his wife mentally or emotionally and/or to exert control over her. Physical violence was considered when the husband hurts or tries to hurt his wife by hitting, kicking, or using another type of physical force. Sexual violence was defined as forcing or attempting to force his wife to take part in a sex act when she does not or cannot consent [14]. Financial abuse was considered when the husband has control over his wife’s access to economic resources.

The third section of the questionnaire was designed by two authors from the research team after an extensive literature review on IPV and its types. Face validity was confirmed by the rest of the research team. Content validity of the designed questions was then ensured after being judged by a panel of five mental health and public health experts, who had previous expertise in the field of IPV. The questions were assessed for relevance and clarity, some modifications were conducted accordingly based on the feedback of the experts, until agreement on the final form of the questions was confirmed.

### Statistical analysis

SPSS statistical software version 21 was used for the analysis. Descriptive statistics were carried out to describe the characteristics of the study participants. Categorical variables were described using percentages, while quantitative variables were described using mean and standard deviation. To determine if there were differences in the exposure to IPV before and during the lockdown, McNemar’s test was used. Binary logistic regression analysis was performed to identify the independent predictors of exposure to IPV during the COVID-19 lockdown (exposed = 1, not exposed = 0). The cutoff value for significance was set at p value < 0.05.

## Results

The age of the participants ranged from 18 to 68 years with a mean of 35.2 ± 7.8 years. The highest percent of them (44.4%) aged from 25 to < 35 years followed by those aged 35 to < 45 years (35.3%). More than half of the participants (56.3%) had university education, and 29% had postgraduate education. Less than one-third (29.6%) were housewives, with 12% of the sampled women reported having an insufficient family income. About 53% of women were from Asian countries (Saudi Arabia, United Arab Emirates, Kuwait, Qatar, Oman, Yemen, Palestine, Iraq, Jordon, and Syria) and 47% were from African Arab countries (Egypt, Libya, Sudan, and Morocco). The majority of the participants (88.8%) reported that the COVID-19 lockdown in their country of residence was partial, 17.8% mentioned that their husbands lost their jobs during the lockdown, 40.6% reported that their family income was affected by the lockdown, and 55.1% stated that their husbands stayed all day at home.

**Fig. 1:**
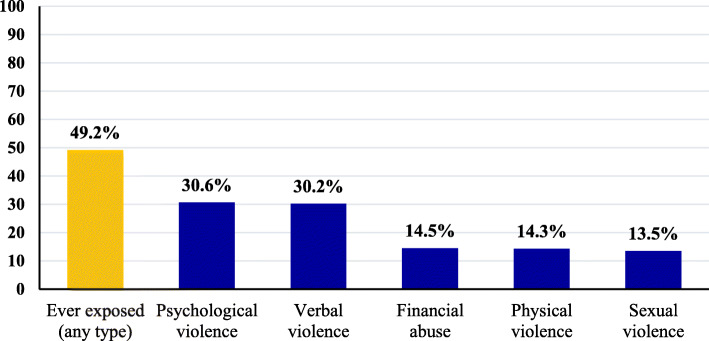
Distribution of adult Arab women according to the types of IPV they were ever exposed to

Figure 1 illustrates that half of women surveyed reported ever exposure to IPV. Psychological violence ranked 1st (30.6%) among the types of IPV to which women were ever exposed to, followed by verbal violence (30.2%), while 14.5%, 14.3%, and 13.5% of women reported that they were exposed to financial abuse, physical, and sexual violence, respectively.

 Table 1 shows that the exposure of women to any type of IPV during the lockdown has significantly increased by 7.3% compared to before the lockdown. The percent of exposure to psychological, physical, and sexual violence has significantly increased during the lockdown compared to before the lockdown, while the increase in the percent of women who reported financial abuse was not significant. Similar proportions of women were exposed to verbal violence before and during the lockdown.

**Table 1:** Distribution of adult Arab women according to their exposure to IPV before and during COVID-19 lockdown

Type of violence	Before lockdown	During lockdown	% change	McNemar p
	No. (%)	No. (%)		
IPV (any type)	194 (39.6)	230 (46.9)	7.3	0.000*
Verbal violence	132 (26.9)	132 (26.9)	0.0	1.000
Psychological violence	100 (20.4)	130 (26.5)	6.1	0.000*
Physical violence	34 (6.9)	64 (13.1)	6.2	0.000*
Sexual violence	44 (9.0)	66 (13.5)	4.5	0.000*
Financial abuse	52 (10.6)	63 (12.9)	2.3	0.052
Mean number of types of violence	0.74 ± 1.11	0.93 ± 1.28		Paired t test = − 6.79 (0.000*)

*Significant (p < 0.05)

The number of different types of IPV to which women were exposed to either before or during the lockdown ranged from 0 to 5 types. The highest percent (19%) were exposed to one type, followed by those exposed to two, three, or more than three types, respectively (11%, 5.9%, and 3.7%, respectively). During the lockdown, 22.2% of women reported that they were exposed to one type of IPV, while women exposed to two, three, or more than three types amounted to 12.4%, 5.5%, and 6.7%, respectively.

A significant increase in the mean number of types of violence to which women were exposed to during the lockdown could also be noticed in Table 1 (from 0.74 ± 1.11 before lockdown to 0.93 ± 1.28 during the lockdown).

The frequency of exposure to the different types of IPV ranged from 1–3 times per month to almost every day, but the most commonly reported was 1–3 times per month (Table 2).

**Table 2:** Distribution of adult Arab women according to the frequency of exposure to different types of IPV during the COVID-19 lockdown

Frequency of exposure to IPV	No. (%) n = 490
**Verbal violence**	
Never	350 (71.4)
1–3 times/month	71 (14.5)
1–2 times/week	38 (7.8)
Most days of the week	31 (6.3)
**Psychological violence**	
Never	414 (84.5)
1–3 times/month	36 (7.3)
1–2 times/week	17 (3.5)
Most days of the week	23 (4.7)
**Physical violence**	
Never	425 (86.8)
1–3 times/month	48 (9.8)
1–2 times/week	6 (1.2)
Most days of the week	11 (2.2)
**Sexual violence**	
Never	424 (86.5)
1–3 times/month	27 (5.5)
1–2 times/week	21 (4.3)
Most days of the week	18 (3.7)
**Financial abuse**	
Never	419 (85.5)
1–3 times/month	46 (9.4)
1–2 times/week	11 (2.2)
Most days of the week	14 (2.9)

Perceived reasons for exposure to IPV included bad husband’s temperament, arguments about decisions concerning family, and financial causes. The correlation between the number of perceived reasons for exposure to IPV and the number of IPV types was tested and it was positive, moderate, and statistically significant (r = 0.53, p = 0.000). Moreover, 18.4% of the women who were exposed to any type of IPV during lockdown reported suffering from psychological problems, while 8.7% reported that the violence resulted in injuries. On the other hand, 42.7% of exposed women did not ask for help after their exposure.

Table 3 shows that the level of education, region of residence, family income, a husband who lost his job, and affection of the family income were the factors significantly associated with exposure to any type of IPV during the lockdown. Higher level of education was associated with less exposure to psychological, physical, and sexual violence. Also being from Asia was associated with less exposure to verbal, psychological, and physical violence. On the other hand, women exposed to all types of IPV, except sexual, were more likely to have an insufficient family income compared to unexposed women. Being a housewife was associated with verbal and psychological violence. Women exposed to verbal, psychological, and physical violence, and financial abuse had more probability of having a husband who lost his job during the lockdown. Having a family income affected by lockdown was associated with verbal, psychological, and physical violence.

Table 3 also shows that all perceived reasons of IPV had a significant association with the exposure to any type of IPV during lockdown. Husband’s temperament and financial reasons were significantly associated with exposure to all types of violence during lockdown.

**Table 3:** Crude odds ratio of the different types of IPV among adult Arab women during lockdown and their personal and social characteristics and the perceived reasons for exposure

Variables	Type of violence Crude odds ratio (95% confidence interval)					
	Verbal violence	Psychological violence	Physical violence	Sexual violence	Financial abuse	Any type of IPV
**Personal and social factors**						
Women’s age (35 years and above vs < 35 years)	1.22 (0.82–1.81)	1.03 (0.69–1.54)	1.27 (0.75–2.15)	1.03 (0.61–1.72)	1.14 (0.67–1.93)	1.1 (0.77–1.56)
Level of education (higher vs 2ry and below)	0.65 (0.38–1.10)	0.41 (0.24–0.69)*	0.28 (0.15–0.51)*	0.43 (0.23–0.79)*	0.62 (0.32–1.20)	0.55 (0.33–0.91)*
Region of residence (Asia vs Africa)	0.56 (0.38–0.84)*	0.45 (0.29–0.68)*	0.23 (0.12–0.42)*	0.82 (0.49–1.37)	0.91 (0.54–1.54)	0.51 (0.35–0.73)*
Nature of lockdown (total vs partial)	0.92 (0.48–1.74)	0.51 (0.24–1.07)	0.64 (0.24–1.67)	0.47 (0.17–1.35)	1.18 (0.53–2.62)	0.86 (0.49–1.51)
Work status (housewife vs employed)	1.69 (1.12–2.58)*	1.74 (1.14–2.66)*	1.51 (0.87–2.62)	1.22 (0.71–2.13)	1.44 (0.83–2.50)	1.31 (0.89–1.94)
Family income (not enough vs others)	3.62 (2.07–6.32)*	3.42 (1.96–5.98)*	3.67 (1.95–6.92)*	1.78 (0.89–3.57)	3.39 (1.78–6.43)*	3.19 (1.76–5.79)*
Husband lost his job (yes vs no)	1.99 (1.23–3.25)*	2.79 (1.71–4.51)*	2.66 (1.49–4.78)*	1.44 (0.77–2.69)	1.89 (1.02–3.48)*	2.69 (1.65–4.37)*
Family income affected by lockdown (yes vs no)	1.69 (1.13–2.53)*	1.69 (1.13–2.53)*	1.79 (1.06–3.04)*	0.88 (0.51–1.49)	1.39 (0.82–2.36)	1.59 (1.11–2.28)*
Duration husband stays at home (all day vs night only)	1.06 (0.71–1.58)	0.93 (0.62–1.39)	1.53 (0.89–2.65)	1.39 (0.82–2.38)	0.66 (0.39–1.12)	1.08 (0.75–1.54)
**Perceived reasons:**						
Bad temperament	19.65 (10.57–36.52)*	5.62 (3.35–9.41)*	10.36 (5.77–18.60)*	4.86 (2.74–8.64)*	5.29 (2.96–9.48)*	29.17 (10.45–81.38)*
Management decisions concerning family	8.41 (4.55–15.54)*	2.75 (1.56–4.86)*	1.76 (0.86–3.61)	1.27 (0.59–2.72)	3.68 (1.93–7.02)*	14.53 (5.69–37.13)*
Financial reasons	7.31 (3.81–14.05)*	6.05 (3.21–11.43)*	4.77 (2.45–9.31)*	2.81 (1.39–5.67)*	9.66 (5.00–18.64)*	20.27 (6.19–66.26)*
Bad circumstances at the husband's work	3.18 (1.61–6.26)*	2.88 (1.46–5.69)*	2.33 (1.04–5.19)*	4.09 (1.96–8.51)*	1.65 (0.69–3.94)	8.24 (3.15–21.54)*
Family members or friends involvement	3.29 (1.08–9.96)*	4.66 (1.49–14.50)*	3.09 (0.92–10.34)	1.97 (0.53–7.36)	2.09 (0.56–7.79)	6.48 (1.42–29.55)*
Arguments about wife’s job	5.64 (1.39–22.87)*	2.25 (0.59–8.53)	1.93 (0.39–9.51)	1.86 (0.38–9.16)	5.72 (1.49–21.91)*	9.33 (1.16–75.20)*

*Significant (p < 0.05)

Three independent factors were found to predict the exposure to any type of IPV during the COVID-19 lockdown as shown in Table 4. The first was the country of residence. Women exposed to IPV were about 2 times more likely to be from African countries compared to unexposed women. The second and third factors were family income and whether the husband lost his job during lockdown. Women exposed to IPV were 56% and 48% less likely to have sufficient family income and to have husbands who did not lose their jobs during lockdown compared to unexposed women, respectively. The model correctly predicted 61% of cases.

**Table 4:** Logistic regression analysis for the predictors of IPV among adult Arab women during COVID-19 lockdown

Independent variables	Odds ratio	p value	95% confidence interval	Sensitivity of the model
Region of residence (Africa)	1.87	0.001*	1.27–2.75	61.0%
Family income (enough)	0.44	0.018*	0.23–0.87	
Husband lost his job (no)	0.52	0.026*	0.29–0.92	

*Significant (p < 0.05)

Variables used to build the model: age, working status, country of residence, family income, nature of lockdown, affection of the family income by lockdown, duration husbands stay at home, husband lost his job during lockdown

Linear regression analysis of the factors predicting the number of IPV types to which adult Arab women exposed to during the COVID-19 lockdown demonstrates that being from Asia and higher education were associated with less number of types of IPV, while insufficient family income was associated with higher number of types of IPV (Table 5), and 12% of the variability in the dependent factor was attributed to these three factors (r^2^ = 0.12, F = 13.1, p = 0.000).

**Table 5:** Linear regression analysis of the factors predicting the number of IPV types to which adult Arab women exposed to during the COVID-19 lockdown

Independent variables	B	Standard error	p value
Region of residence (Asia)	− 0.49	0.14	0.000*
Family income (not enough)	0.87	0.23	0.000*
Level of education (higher education)	− 0.56	0.19	0.003*
Constant	2.72	0.44	0.000*
	r^2^ = 0.12	F = 13.1	p = 0.000*

*Significant (p<0.05)

Variables used to build the model: level of education, family income, husband lost his job during lockdown, family income affected by lockdown

## Discussion

IPV is a deeply rooted public health problem in the Arab world that is expected to be on rise following the COVID-19 pandemic [6, 15]. Being one of the hidden consequences of the lockdown, IPV has been increasingly reported as a public health issue of concern [16, 17]. The current work investigated IPV among a sample of Arab married women during the COVID-19 lockdown. Ever exposure to any type of IPV was recorded by half of women surveyed (49.2%). This figure is higher than the rates of lifetime exposure to physical or sexual violence reported by the WHO for ever-partnered women worldwide and in the Eastern Mediterranean region (30% and 37%, respectively) [3] and can be explained by the inclusion of other forms of IPV in the current study. Current findings were in line with those reported by Elghossain et al. (2019) [15], in their systematic review on the prevalence of IPV in the Arab world. The reported rates of ever exposure reached up to 59% for physical violence; 40% for sexual violence; and the highest was the psychological violence (up to 91%).

A significant increase in the women’s exposure to any IPV during the lockdown by 7.3% was found, specifically for the physical and sexual violence, as well as the psychological abuse behaviors including withhold from health care and preventing contact with family. Although similar studies are scarce, yet, different worldwide reports from the WHO and countries such as China, Italy, Brazil, France, Germany, the UK, the USA, Australia, Lebanon, and Malaysia indicate the escalation of IPV during COVID-19 lockdown [16, 18, 19]. Preliminary data from a similar online survey in New Orleans indicated a 59% increase in IPV among those who experienced IPV before the COVID-19 crisis [20]. In addition, Gosangi et al. (2020) [21] reported an increase in the incidence of physical IPV during the COVID-19 pandemic by 1.8 times compared to 2017–2019 rates. Current findings were consistent with those from previous outbreaks and natural disasters such as after Hurricane Katrina [22], the outbreaks of Ebola in West Africa [23], and cholera [24].

In this study, living in an African country increased the risk of exposure to IPV by two times, while the sufficient family income and keeping the husband’s job during lockdown reduced the likelihood of exposure by nearly half. These predictors can be interpreted in view of several explanations, the most important one is reflecting the socioeconomic status. Arab African countries included in this study are generally of lesser socioeconomic living standards compared with the Arab Asian countries [25]. Meanwhile, the socioeconomic status is reflected directly in the family income and indirectly by losing the husband’s job. Other explanations include the psychological impact of losing jobs, which may be expressed as violent behavior by the husband, as well as the differences between the two continents in traditions, cultural perception of violence against women, and the level of women empowerment [26–28]. Interestingly, all factors associated independently with different types of IPV were reflecting the socio-economic status of the women as well. These findings were in line with Davis et al. (2020) [29], who found that people who lost their jobs during COVID-19 were 2.5–3 times more likely to perpetrate psychological and physical IPV. The role of economic status in the escalation of IPV during the lockdown is supported by findings from studies conducted during the Great Recession (2007–2015) that found extraordinary increase in domestic violence rates during the recession [30].

The severity of the economic impact of the lockdown was indicated by the finding that the insufficient family income did not only increase the likelihood of exposure, but also the exposure to a higher number of IPV types. In addition, a significant positive correlation between exposure to more IPV types and the number of perceived reasons for exposure was noticed, revealing that the bad husband’s temper and financial causes were the main reasons correlated with almost all types of IPV. The economic burden of the lockdown exerted its impact on the financial situation as well as the husband’s temper leading to exacerbation of the problem. On the other hand, women from Asia and with higher education were exposed to a lesser number of types. Taking into consideration the higher socioeconomic status of women from Arab Asian countries [25], these results were consistent with previously reported risk factors of IPV including the low socioeconomic status and the lower educational level [27, 31].

Noteworthy, nearly half of exposed women (42.7%) did not ask for any kind of help. Several factors may interplay to explain this finding, including women’s acceptance of violence and perceiving it as a husband’s right, the severity of the consequences, and the kind of help needed; whether medical help for physical and psychological consequences or asking for non-professional help in case of husband’s control strategies to isolate the woman. The lockdown may increase women isolation and prevent them from seeking help, as interpreted by Bellizzi et al. (2020) [5], based on reports from Italy indicating that only one quarter of women exposed to violence could reach help during the pandemic.

### Limitations of the study

The sensitivity of approaching such issues like IPV in the Arab world was associated with a huge amount of resistance that faced the researchers during the conduction of this study. In addition, the study findings should be interpreted in light of some limitations such as using self-reported data from a cross-sectional online survey, which also minimizes access to different socioeconomic strata in the community. Use of non-standardized comprehensive tool for assessment of different types of IPV is also found to be a limitation of this study. Further large-scale studies are needed to give a more comprehensive data on IPV in the Arab region. A qualitative approach with face-to-face interviewing technique is recommended.

## Conclusions

The current work unveiled the rising surge of IPV in the Arab world during COVID-19 pandemic lockdown, and its strong association with the socioeconomic consequences of the pandemic on families. Given the fact that recurrence of lockdown is very possible in light of absence of confirmed vaccines and treatment for the virus, and that many families will be suffering from the long-lasting economic consequences of the pandemic, actions towards raising awareness about the problem among professionals and the community, early detection and provision of appropriate services are mandatory.

## Data Availability

All relevant data are available and included in the paper. The datasets used and/or analyzed during the current study are available from the corresponding author on reasonable request.

## Abbreviations

IPV: Intimate partner violence
WHO: World Health Organization
